# Circulating CD36 and oxLDL levels are associated with cardiovascular risk factors in young subjects

**DOI:** 10.1186/1471-2261-14-54

**Published:** 2014-04-28

**Authors:** Luz E Ramos-Arellano, José F Muñoz-Valle, Ulises De la Cruz-Mosso, Aralia B Salgado-Bernabé, Natividad Castro-Alarcón, Isela Parra-Rojas

**Affiliations:** 1 Laboratorio de Investigación en Obesidad y Diabetes, Unidad Académica de Ciencias Químico Biológicas, Universidad Autónoma de Guerrero, Chilpancingo, Guerrero, México; 2 Departamento de Biología Molecular y Genómica, Instituto de Investigación en Ciencias Biomédicas, Centro Universitario de Ciencias de la Salud, Universidad de Guadalajara, Guadalajara, Jalisco, México

**Keywords:** CD36, OxLDL, Cardiovascular risk factors, Obesity

## Abstract

**Background:**

Cardiovascular disease (CVD) results from a combination of abnormalities in lipoprotein metabolism, oxidative stress, chronic inflammation, and susceptibility to thrombosis. Atherosclerosis is the major cause of CVD. CD36 has been shown to play a critical role in the development of atherosclerotic lesions by its capacity to bind and promote endocytosis of oxidized low-density lipoprotein (oxLDL) and is implicated in the formation of foam cells. The purpose of this research was to evaluate whether there is an association of sCD36 and oxLDL levels with cardiovascular risk factors in young subjects.

**Methods:**

A total of 188 subjects, 18 to 25 years old, 133 normal-weight and 55 obese subjects from the state of Guerrero, Mexico were recruited in the study. The lipid profile and glucose levels were measured by enzymatic colorimetric assays. Enzyme-linked immunosorbant assays (ELISA) for oxLDL and sCD36 were performed. Statistical analyses of data were performed with Wilcoxon- Mann Whitney and chi-square tests as well as with multinomial regression.

**Results:**

TC, LDL-C, TG, oxLDL and sCD36 levels were higher in obese subjects than in normal-weight controls, as well as, monocyte and platelet counts (*P* < 0.05). Obese subjects had 5.8 times higher risk of sCD36 in the third tertil (>97.8 ng/mL) than normal-weight controls (*P* = 0.014), and 7.4 times higher risk of oxLDL levels in third tertile (>48 U/L) than control group. The subjects with hypercholesterolemia, hypertriglyceridemia, fasting impaired LDL-C had a higher risk of oxLDL levels in the third tertile (>48 U/L) than the control group (*P* < 0.05).

**Conclusions:**

Circulating CD36 and oxLDL levels are associated with cardiovascular risk factors in young subjects and may be potential early markers for cardiovascular disease (CVD).

## Background

Cardiovascular disease (CVD) risk factors such as advanced age, obesity, smoking, hyperlipidemia, diabetes, and hypertension, account for 30–40% of the worldwide prevalence of this disease [1]. The pathological basis of CVD results from a combination of abnormalities in lipoprotein metabolism, oxidative stress, chronic inflammation, and susceptibility to thrombosis [2]. Atherosclerosis is the major cause of CVD [3], it is considered a chronic inflammatory disease of the arterial wall that underlies many of the common causes of cardiovascular morbidity and mortality, including myocardial infarction (MI), cerebrovascular and peripheral vascular disease [4].

A key process in the pathogenesis of atherosclerosis is the deposition of cholesterol in the arterial wall. Lipoproteins are involved in this process, including cholesterol carried by very low-density (VLDL), remnant lipoproteins and low-density lipoproteins (LDL), particularly the small and dense forms; conversely, cholesterol is carried away from the arterial wall by high-density lipoprotein (HDL) [5].

Oxidative modification of LDL (oxLDL) in the arterial wall is central to the pathogenesis of atherosclerosis [6]. OxLDL is associated with carotid intimal-media thickness, unstable plaques in the coronary and carotid arteries, impaired brachial and coronary endothelial function, and coronary artery disease [7]. When oxLDL loses its ability to bind to LDL receptors, this interferes with its normal processing and as a result gains affinity for a family of proteins called scavenger receptors [8]. This leads to macrophage activation, foam-cell formation, secretion of growth factors and proinflammatory cytokines, thereby promoting plaque formation [9,10].

CD36, an 88 kDa glycoprotein, was originally described as platelet receptor glycoprotein which belongs to the class B scavenger receptor family [11,12]. CD36 is expressed on an extensive range of cells and tissues, including microvascular endothelial cells, monocytes and macrophages, dendritic cells, adipocytes, keratinocytes, cardiac and skeletal muscle, retinal pigment epithelium, microglia, reticulocytes, breast, gut, renal epithelium, platelets, hepatocytes, smooth muscle cells and binds to a diverse array of ligands [13-16]. CD36 is best characterized as a free fatty acid transporter involved in different biological processes like angiogenesis, inflammation, lipid metabolism, atherosclerosis and platelet activation [17].

Monocyte/macrophage CD36 has been shown to play a critical role in the development of atherosclerotic lesions by its capacity to bind and promote endocytosis of oxLDL, and is also implicated in the formation of foam cells [18,19]. Moreover, the pathogenic role of oxLDL in atherosclerosis largely depends on CD36 [20].

A soluble form of CD36 (sCD36), a marker of altered tissue CD36 expression, was recently identified in human plasma, and elevated levels were found in obesity and type 2 diabetes. In this regard, sCD36 in plasma has been reported up to 4-fold higher in obese T2D-patients compared to lean healthy control subjects [21]. The circulating concentration of CD36 is also associated with markers of liver injury in subjects with altered glucose tolerance [22]. A recent study revealed that soluble CD36 in plasma correlates significantly with markers of atherosclerosis, insulin resistance and fatty liver in a non-diabetic healthy population [23]. Due to the wide-spread tissue expression of CD36 and its broad range of functions it is difficult to foresee which specific pathological processes may reflect alterations in sCD36 [24]. However, a recent study showed that sCD36 is not a proteolytic product, but it is associated with a specific subset of circulating microparticles (MPs) that can readily be analyzed and which originate mainly from platelets in normal subjects [25]. In this regard, the aim of this research was to evaluate whether there is an association of sCD36 and oxLDL levels with cardiovascular risk factors in young subjects.

## Methods

### Subjects

A total of 188 subjects were randomly selected, 18 to 25 years old, 133 normal-weight controls (BMI 18.5 to 24.9 kg/m2) and 55 obese subjects (BMI ≥30 kg/m2) from the state of Guerrero, Mexico. There were 117 women and 71 men; participants were not under any medication or had evidence of metabolic disease other than obesity. All subjects gave their written informed consent previous explanation of the purpose and nature of the study. The protocol was approved by the Research Ethics Committee of the University of Guerrero.

### Blood pressure

Blood pressure was measured in the sitting position with the use of an automatic sphygmomanometer on the left arm after 10 min rest. The systolic blood pressure (SBP) and diastolic blood pressure (DBP) were calculated from two readings with a minimal interval of 10 min. Hypertension was defined as mean SBP ≥140 mmHg and/or DBP ≥90 mmHg [26].

### Biochemical analysis

A venous blood sample of 5 mL was obtained from each subject after at least a 12 hours fasting. All serum lipid levels and glucose were determined by enzymatic methods with commercially available kits (Spinreact). Abnormal biochemical levels were identified when total-cholesterol (TC) ≥ 200 mg/dL, triglycerides (TG) ≥ 150 mg/dL, low-density lipoprotein cholesterol (LDL-C) > 100 mg/dL, high-density lipoprotein cholesterol (HDL-C) < 40 mg/dL and glucose >100 mg/dL, based on the criteria of the National Cholesterol Education Program (NCEP) Expert Panel on Detection, Evaluation, and Treatment of High Blood Cholesterol in Adults (Adult Treatment Panel III) [27].

### Determination of sCD36 and oxLDL levels

Enzyme-linked immunosorbant assays (ELISA) for oxLDL (Mercodia Oxidized LDL ELISA) and sCD36 (Human soluble CD36 ELISA, kit-Aviscera Bioscience) were performed, according to the manufacturer’s instructions, with an intra-assay CV <6% and interassay CV <7% for oxLDL ELISA assay, and an intra-assay CV <5% and interassay CV <9% for CD36 ELISA assay.

### Statistical analysis

The statistical analyses were performed with the statistical software package SPSS 15.0 and STATA software 9.0. Quantitative variables were expressed as medians and 25th to 75th percentiles and significant differences between groups were determined using the Wilcoxon-Mann Whitney test. Qualitative variables were expressed as percentages and the differences between groups were determined using the chi-square test. The association analysis between serum levels of oxLDL and sCD36 with lipid phenotypes was carried out with multinomial regression, where a *P* value <0.05 was considered statistically significant.

## Results

### General and biochemical characteristics

General and biochemical characteristics of the study subjects are shown in Table 1. Measurements of body weight, height, systolic and diastolic blood pressure, prevalence of hypertension, and TC, LDL-C, TG, sCD36 and oxLDL levels were higher in obese subjects than in normal-weight controls, as well as, the monocyte and platelet counts (*P* < 0.05). There were no significant differences by gender (*P* = 0.162).

**Table 1:** Anthropometric and biochemical variables by group

**Variables**	**Normal weight**	**Obesity**	***P *** **value**
n	133	55	-
Age (years)	20 (19-22)	21 (20-23)	0.005
Gender			0.162
Female (%)	87 (65)	30 (55)	
Male (%)	46 (35)	25 (45)	
Weight (kg)	54.5 (49.2-59.4)	90.9 (79.1-99.9)	0.001
Height (cm)	158.7 (153.0-167.5)	163.5 (155-171)	0.036
BMI (kg/m^2^)	21.5 (20.0-23.4)	33.1 (31.3-35.3)	0.001
SBP (mmHg)	104 (98-112)	115 (112-121)	0.001
DBP (mmHg)	68 (61-73)	70 (67-77)	0.003
Hypertension			0.001
No	124 (93.9)	35 (67.3)	
Yes	8 (6.1)	17 (32.7)	
TC (mg/dL)	152 (136-173)	160 (137-195)	0.057
HDL-C (mg/dL)	43 (38-54)	44 (38-47)	0.717
LDL-C (mg/dL)	89 (61-116)	117 (88-157)	0.001
TG (mg/dL)	72 (56-98)	121 (75-89)	<0.001
Glucose (mg/dL)	83 (75-89)	83 (77-90)	0.386
Monocytes (%)	7 (5-9)	9 (7-11)	0.0005
Platelets (10^3^/mm^3^)	247 (215-289)	265 (232-307)	0.048
sCD36 (ng/mL)	32.3 (16.8-102.4)	146.3 (76.7-694.7)	0.002
oxLDL (U/L)	35.4 (27.9-47.1)	51.5 (38.0-59.7)	<0.001

BMI, Body Mass Index; SBP, Systolic Blood Pressure; DBP, Diastolic Blood Pressure; TC, Total Cholesterol; HDL-C, High-Density Lipoprotein Cholesterol; LDL-C, Low-Density Lipoprotein Cholesterol; TG,Triglyceride; oxLDL, oxidized Low-Density Lipoprotein. Values were presented as median (percentile 25-75th). Difference between genders was determined by the Wilcoxon-Mann–Whitney test. Hypertension data were presented in (n) and percentages; the difference between genders was determined by chi-square test.

### Correlations between sCD36 and oxLDL levels with selected variables

In all subjects studied, sCD36 correlated significantly with weight, BMI, waist, hip, waist-to-hip ratio,% fat,% fat mass, LDL-C, oxLDL and monocyte count. Obese subjects showed a high correlation among BMI and sCD36 (r = 0.50, *P* = 0.028) (Table 2). The oxLDL levels correlated positively with weight, BMI, waist, hip, waist-to-hip ratio,% fat,% fat mass, and TC, TG, LDL-C, sCD36 and monocyte count in all subjects. In normal-weight and obese groups, oxLDL levels showed a high correlation with weight, BMI, waist, waist-to-hip ratio,% fat mass, and TG, LDL-C levels and monocyte count (Table 3).

**Table 2:** Correlation between sCD36 and selected variables

**Variables**	**All subjects**		**Normal weight**		**Obesity**	
	**r**	***p***	**r**	***p***	**r**	***p***
Weight (kg)	0.29	0.004	0.14	0.217	0.41	0.073
Height (cm)	0.03	0.774	0.04	0.724	0.15	0.534
BMI (kg/m^2^)	0.32	0.001	0.17	0.150	0.50	0.028
Waist (cm)	0.30	0.003	0.13	0.2474	0.30	0.210
Hip (cm)	0.31	0.002	0.18	0.1190	0.30	0.201
Waist-to-hip ratio	0.23	0.026	0.001	0.988	0.02	0.931
% Fat	0.32	0.001	0.19	0.097	-0.02	0.909
% Fat mass	0.35	0.0006	0.22	0.058	0.30	0.203
Glucose (mg/dL)	-0.15	0.132	-0.16	0.168	-0.01	0.954
TC (mg/dL)	-0.05	0.609	-0.15	0.201	0.16	0.479
TG (mg/dL)	0.15	0.134	-0.001	0.991	0.33	0.151
HDL-C (mg/dL)	0.07	0.456	0.10	0.373	-0.06	0.771
LDL-C (mg/dL)	0.21	0.038	0.02	0.803	0.23	0.325
oxLDL (U/L)	0.25	0.024	0.06	0.623	0.29	0.334
Monocytes (%)	0.33	0.001	0.22	0.056	0.20	0.376
Platelets (10^3^/mm^3^)	0.15	0.142	0.03	0.741	0.23	0.309

r = Spearman correlation coefficient; *p* = *p* value.

**Table 3:** Correlation between oxLDL levels and selected variables

**Variables**	**All subjects**		**Normal weight**		**Obesity**	
	**r**	***p***	**r**	***p***	**r**	***p***
Weight (kg)	0.43	0.0001	0.23	0.009	0.31	0.026
Height (cm)	0.05	0.438	-0.06	0.499	0.17	0.234
BMI (kg/m^2^)	0.51	0.0001	0.40	<0.001	0.37	0.009
Waist (cm)	0.52	0.0001	0.39	<0.001	0.38	0.007
Hip (cm)	0.36	0.0001	0.10	0.225	0.21	0.139
Waist-to-hip ratio	0.53	0.0001	0.42	<0.001	0.30	0.034
% Fat	0.32	0.0001	0.13	0.124	0.09	0.516
% Fat mass	0.41	0.0001	0.21	0.014	0.39	0.005
Glucose (mg/dL)	-0.10	0.182	-0.19	0.027	0.12	0.398
TC (mg/dL)	0.32	<0.001	0.17	0.053	0.67	<0.001
TG (mg/dL)	0.47	<0.001	0.38	<0.001	0.33	0.018
HDL-C (mg/dL)	0.04	0.557	0.04	0.621	0.11	0.422
LDL-C (mg/dL)	0.43	<0.001	0.33	0.0001	0.53	0.0001
sCD36 (ng/mL)	0.25	0.024	0.06	0.623	0.29	0.334
Monocytes (%)	0.40	<0.001	0.31	0.0003	0.34	0.020
Platelets (10^3^/mm^3^)	0.01	0.889	0.01	0.879	-0.15	0.293

r = Spearman correlation coefficient; *p* = *p* value.

### Circulating CD36 and oxLDL levels according to metabolic abnormalities

Subjects with hypertriglyceridemia and hypertension had higher levels of sCD36, while oxLDL levels were higher in subjects with hypercholesterolemia, hypertriglyceridemia, LDL-C and hypertension compared to those subjects without these abnormalities (*P* < 0.05) (Table 4).

**Table 4:** sCD36 and oxLDL levels according to metabolic abnormalities

**Variables (n)**	**sCD36 levels (ng/mL)**	**oxLDL levels (U/L)**
Fasting glucose (>100 mg/dL)		
No (176)	47.7 (18.4-219.9)	37.8 (29.5-50.6)
Yes (12)	62.2 (16.7-200.4)	52.7 (30.1-60.5)
	*P* = 0.93	*P* = 0.20
Hypercholesterolemia (≥200 mg/dL)		
No (162)	44.9 (17.3-223.6)	36.7 (29.1-48.5)
Yes (26)	74.8 (25.7-98.7)	54.5 (45.5-65.0)
	*P* = 0.55	*P* = 0.0002
Fasting LDL-C (>100 mg/dL)		
No (95)	47.7 (17.2-208.2)	32.4 (27.8-45.6)
Yes (93)	74.8 (21.8-270.4)	47.1 (35.8-57.1)
	*P* = 0.30	*P* = 0.0001
Fasting HDL-C (<40 mg/dL)		
No (88)	61.1 (19.6-262)	45.6 (31.9-54.3)
Yes (100)	46.7 (17.9-127.2)	34.6 (29-48.5)
	*P* = 0.49	*P* = 0.06
Hypertriglyceridemia (≥150 mg/dL)		
No (161)	43.1 (16.7-190.6)	36.1 (28.2-48.1)
Yes (27)	180.3 (66.01-2295)	52.5 (48.5-59.7)
	*P* = 0.01	*P* = 0.0001
Hypertension (SBP ≥140 mmHg, DBP ≥ 90 mmHg)		
No (161)	34.1 (16.7-190.6)	37.6 (29–119.0)
Yes (27)	79.8 (66.01-200.4)	48.0 (31.5-58.8)
	*P* = 0.03	*P* = 0.1218

The values were presented as median (percentile 25-75th). The difference between genders was determined by the Wilcoxon-Mann–Whitney test.

### Association of cardiovascular risk factors with sCD36 and oxLDL levels

For the association analysis with cardiovascular risk factors, sCD36 was classified into tertiles (first tertile <23.3 ng/mL, second tertile 23.3 to 97.8 ng/mL and third tertile >97.8 ng/mL), since there are no established reference values. Obese subjects had 5.8 times higher risk of sCD36 in the third tertile than normal-weight controls, adjusted for age and gender (*P* = 0.014) (Table 5). The oxLDL levels were also classified into tertiles (first tertile <31.9 U/L, second tertile 31.9 to 48.0 U/L and third tertile >48.0 U/L) in order to analyze their association with cardiovascular risk factors. Table 5 shows that subjects with hypercholesterolemia had 7.5 times higher risk of oxLDL levels in the third tertile than subjects without these abnormalities. Individuals with impaired fasting LDL-C had 4.5 times higher risk of oxLDL levels in the third tertile and subjects with hypertriglyceridemia had 17.9 times higher risk of oxLDL levels in the third tertile, adjusted for age, gender and BMI. Likewise, obese individuals had 7.4 times higher risk of oxLDL levels in third tertile than controls, adjusted for age and gender.

**Table 5:** Association of cardiovascular risk factors with sCD36 and oxLDL levels

**Variables**	**sCD36 levels (>97.8 ng/mL)**			**oxLDL levels (>48 U/L)**		
	**OR (95% CI)**	***R***^***2***^	***p value***	**OR (95% CI)**	***R***^***2***^	***p value***
Hypercholesterolemia	0.9 (0.14-5.75)	0.098	0.932*	7.5 (1.80-31.15)	0.155	0.006*
Impaired fasting LDL-C	0.7 (0.22-2.45)	0.085	0.622*	4.5 (1.79-11.31)	0.152	0.001*
Impaired fasting HDL-C	0.4 (0.15-1.46)	0.103	0.195*	0.3 (0.10-0.63)	0.147	0.003*
Hypertriglyceridemia	5.3 (0.50-55.87	0.094	0.164*	17.9 (2.03-158.92)	0.154	0.009*
Hypertension	4.5 (0.45-44.63)	0.098	0.197*	0.51 (0.13-1.98)	0.117	0.336*
Obesity	5.8 (1.43-24.05)	0.070	0.014**	7.4 (2.82-19.60)	0.80	<0.001**

*Adjusted for age, gender and BMI, **Adjusted for age and gender.

## Discussion

In this research, we found a higher prevalence of hypertension in obese subjects (32.7%) than in normal weight subjects (6.1%), with a prevalence of 13.6% in the total study subjects. These findings are similar to those reported in another study in U.S. teenagers; where hypertension showed a prevalence of 30% in obese subjects [28]. It is known that the increase sodium reabsorption induced by angiotensin II produced by adipocytes affects renal natriuresis, so that obese subjects need higher blood pressure levels than normal weight subjects to maintain a balance between the sodium intake and renal diuresis [29].

In this study, as in previous studies the lipid profile (TC, LDL-C and TG) was higher in obese subjects than normal-weight controls. This may be due to increased adiposity since adipose tissue undergoes morphological and physiological changes which include the release of proinflammatory cytokines such as tumour necrosis factor alpha (TNF-α), and in turn, decrease insulin sensitivity and increase lipolysis. These morphological changes contribute to insulin resistance and dyslipidemia [30].

An interesting finding in this study was that obese subjects have a higher number of monocytes and platelets than the normal-weight subjects. The leukocyte count is considered as an indicator of inflammatory status in obesity [31]. In addition, research has shown that adults and children with obesity have higher levels of leukocytes, mainly monocytes, compared with adults and children of normal weight [32-34]. Regarding increased platelet count in subjects with obesity, similar findings have been shown in other studies, where the platelet count is higher in teenagers and adults with obesity than normal weight subjects [35,36]. In relation to cytokines, it has been shown that interleukin-6 (IL-6) induces differentiation of megakaryocytes into platelets and that IL-6 is produced by adipose tissue [37,38]. Furthermore, it has been reported that obese individuals have increased levels of IL-6 [39], which may explain the increase in platelets in an obese state.

In this study, we observed that sCD36 was higher in obese subjects than in normal weight subjects (143.3 ng/mL vs. 32.3 ng/mL, *P* = 0.002), these results are congruent with previous studies [21,40]. This may be due to the increased number of platelets and monocytes shown in obese subjects, as was recently reported that the circulating form of the CD36 receptor is associated with microparticles mainly originated of platelets, leukocytes and endothelial cells as a result of stimuli or apoptosis [8,25]. These microparticles have been found increased in subjects with insulin resistance and obese with type 2 diabetes, due to presence of a low-grade inflammation [41-43]. In this study, we observed that serum oxLDL levels were higher in obese subjects than in control group (51.5 U/L vs. 35.4 U/L), these results are consistent with those reported in other studies [44]. This may be due to the increase oxidative stress in an obese state, which favors the oxidation of LDL-C [45,46].

We observed a high correlation of sCD36 with BMI in obese subjects (r =50, *P* = 0.028), similar results have been reported in previous studies [47]. In addition, oxLDL levels showed a strong correlation with BMI, TG and LDL-C in subjects with and without obesity. Such correlations are similar to those reported in other studies [48,49].

In this research, sCD36 in the third tertile (>97.8 ng/mL) were associated with obesity, although there is a lack of studies that support this association, sCD36 have been correlated with BMI [47,50]. Higher sCD36 in obese subjects than in normal weight subjects have also been reported in other studies [21,43]. Furthermore, it is proposed that high CD36 levels may be a marker of increased CD36 expression known from a number of tissues that are associated with the metabolic syndrome; macrophage infiltration and low-grade inflammation in abdominal obesity, which may lead to dyslipidemia and peroxidation of lipoproteins [24].

We also found that oxLDL levels in third tertile (>48.0 U/L) were associated with hypercholesterolemia, impaired fasting LDL-C, hypertriglyceridemia and obesity. The association between dyslipidemia and oxidation of LDL has been demonstrated in individuals in the pre-diabetic state [48]. It has also been observed in middle-aged people that obesity and dyslipidemia are the strongest predictors of oxLDL levels [51]. The association between cardiovascular disease (CVD) and oxLDL has been demonstrated in others studies [52-55]. Considering the associations shown in this study (sCD36 and oxLDL levels with traditional cardiovascular risk factors such as obesity, hypercholesterolemia, hypertriglyceridemia and impaired fasting LDL-C), measuring sCD36 and oxLDL levels can be incorporated into cardiovascular risk factors in young subjects for early diagnosis of cardiovascular disease.

Our research has some limitations. We could not study the associations between sCD36 and oxLDL levels with early atherosclerosis. As our study is comprised of young subjects without clinical atherosclerotic diseases, we were only able to study associations between traditional cardiovascular risk factors with sCD36 and oxLDL levels. Whether the increase of these markers in young subjects is associated with the silent phase of atherosclerosis remains to be elucidated. The authors believe that a greater obese group is desirable to improve the power of the study.

## Conclusions

In this research, sCD36 and oxLDL levels are associated with cardiovascular risk factors, particularly with obesity, hypercholesterolemia, impaired fasting LDL-C and HDL-C and hypertriglyceridemia. Therefore, sCD36 and oxLDL levels may be potential early markers for CVD. However, these associations should be investigated in further studies.

## Competing interests

The authors declare that they have no competing interests.

## Authors’ contributions

LERA performed ELISA assays, statistical analysis and writing the manuscript. UDCM carried out the immunoassays. ABSB performed biochemical measurements and quality control. JFMV and NCA participated in the critical revision of the manuscript. IPR conceived the study and participated in manuscript preparation. All authors read and approved the final manuscript.

## Pre-publication history

The pre-publication history for this paper can be accessed here:

http://www.biomedcentral.com/1471-2261/14/54/prepub
